# Comparison Between Nylon and Polyglactin Sutures in Pediatric Cataract Surgery: A Randomized Controlled Clinical Trial

**DOI:** 10.3389/fmed.2021.700793

**Published:** 2021-08-27

**Authors:** Mathias V. Melega, Roberto dos Reis, Rodrigo Pessoa Cavalcanti Lira, Denise Fornazari de Oliveira, Carlos Eduardo Leite Arieta, Monica Alves

**Affiliations:** ^1^School of Medical Sciences, University of Campinas, São Paulo, Brazil; ^2^School of Medical Sciences, Federal University of Pernambuco (UFPE), Recife, Brazil

**Keywords:** pediatric cataract, suture, nylon, polyglactin, suture-related complications

## Abstract

**Purpose:** To compare the performance of nylon sutures to that of polyglactin sutures in pediatric patients undergoing cataract surgery.

**Setting:** University of Campinas (UNICAMP), Campinas, São Paulo, Brazil

**Design:** A prospective, randomized, partially masked, single-site clinical trial. (https://clinicaltrials.gov/ct2/show/NCT03812640).

**Methods:** A total of 80 eyes from 80 patients who underwent pediatric cataract surgery were randomized into two groups in block sizes of four. Group A consisted of 41 patients whose surgical incisions were sutured with polyglactin 10-0 material. Group B consisted of 39 patients whose surgical incisions were sutured with nylon 10-0 material. The primary outcome was frequency of suture-related complications in each group. Secondary outcomes were the frequency with which suture removal was necessary.

**Results:** The incidence of suture-related complications within 6 months of follow up was 0 out of 41 eyes (0.00%) in the polyglactin group and 17 out of 39 eyes (43.59%) in the nylon control group (*p* < 0.001). In all of the eyes with suture-related complications, the sutures were promptly removed. The most frequent complications were vascularization near the suture (17.95%) and loose sutures (17.95%). No ocular or systemic study-related adverse events were observed.

**Conclusions:** Polyglactin sutures were found to be safe and effective for pediatric patients undergoing cataract surgery. Their lower rate of complications and reduced likelihood of removal (and the subsequent need for general anesthesia) make their use preferrable to that of nylon sutures. This study represents the first controlled randomized clinical trial to compare nylon sutures to polyglactin sutures in pediatric patients undergoing cataract surgery.

**Clinical Trial Registration:** URL: https://clinicaltrials.gov/ct2/show/, Identifier: NCT03812640.

## Introduction

Though rare, pediatric cataracts are a major cause of childhood blindness. Children deprived of adequate treatment experience worsened quality of life, and the socioeconomic costs are higher overall. The prevalence of pediatric cataracts is estimated to range from 1:10,000 to 4:10,000 in industrialized nations and from 5:10,000 to 15:10,000 in developing countries. Pediatric cataracts generate ~200,000 cases of blindness in children around the world each year as a result of unoperated cataracts, surgical complications, or consequences of diseases such as glaucoma and amblyopia (1–4).

Pediatric cataracts must be diagnosed as early as possible; a late diagnosis worsens the visual prognosis. Because the patients are children, trauma or eye rubbing in the postoperative period cannot be fully avoided. In addition, children's sclera exhibits limited rigidity, resulting in poor integrity of the surgical incision if no sutures are made. This situation requires surgeons to systematically suture the surgical incision in order to guarantee the perfect closure of the eyeball and prevent complications such as leakage of the aqueous humor with postoperative hypotonia, iris prolapse, or anterior synechiae formation, as well as to prevent intraocular infection caused by the entrance of microorganisms into the incision. In Brazil, this suture has been performed using 10-0 nylon for decades. Because nylon is a non-absorbable material, it can remain in the patient's cornea for years, predisposing the patient to suture loosening with the accumulation of mucus, corneal erosion, corneal neovascularization, infectious keratitis, (5–7) endophthalmitis, (8, 9) and giant papillary conjunctivitis (10). Because of the risk of complications associated with suturing, the removal of these sutures is essentially obligatory, though they must be removed under sedation in a surgical facility (11, 12). Due to the need for nylon suture removal, other materials are being considered.

In 1998, a randomized clinical trial comparing polyglactin 10-0 to nylon 10-0 in adult cataract surgeries with a 5.2 mm incision demonstrated safety in incision closure and a low rate of complications associated with the use of polyglactin; because polyglactin can be absorbed by the body within 56–70 days, additional interventions to remove it are not necessary (13, 14). In 2007, a retrospective study on pediatric patients comparing absorbable polyglactin sutures to non-absorbable polyester sutures demonstrated a lower rate of complications in the patients who received the polyglactin sutures (15). This study found that the polyglactin sutures produced no complications and that their removal was not necessary; meanwhile, the polyester sutures were associated with complications in 18% of the cases in which they were used, and their removal was required. However, the study was retrospective and used vastly different group sizes.

To our knowledge, no controlled randomized clinical trials have been performed to compare sutures performed using nylon to those performed using polyglactin in pediatric patients. We performed the current study in an attempt to establish evidence-based conclusions regarding the prevention of suture-related complications.

Our study is the first randomized clinical trial to compare nylon sutures to polyglactin sutures in terms of rates of complications and the frequency with which suture removal is necessary in pediatric patients undergoing cataract surgery.

## Materials and Methods

This study was a single-site, prospective, parallel-group, randomized, partially masked, phase 3 clinical trial. It was performed after approval from the University of Campinas research ethics committee and was conducted in accordance with the tenets of the Declaration of Helsinki and current legislation on clinical research. Written informed consent was obtained from all subjects after the explanation of the procedures and study requirements. The trial was registered and began in January 2019 (Comparison Between Nylon and Polyglactin Corneal Suture in Pediatric Cataract Surgery: A Randomized and Controlled Clinical Trial; ClinicalTrials.gov identifier: NCT03812640; https://clinicaltrials.gov/ct2/show/NC*T0*3812640). The inclusion criteria were patients 14 years of age or younger for whom pediatric cataract surgery was clinically indicated. The exclusion criteria were traumatic cataract with ocular perforation, cataract surgery associated with other procedures (such as glaucoma filtering surgery, vitreoretinal surgery, or corneal surgery), signs of ocular or periocular infection, advanced glaucoma, and severe ocular surface disease.

Data were collected from patients undergoing cataract surgery at the University of Campinas (UNICAMP) Clinical Hospital. Medical records, routine preoperative clinical exam data, data from intraoperative evaluations, and data from the 1st, 7th, 30th, 90th, 120th, and 180th postoperative days were collected on each patient.

Patients were randomly divided into one of two groups approximately equal in size and stratified by age (0–6 months, 6–12 months, 1–3 years, 3–6 years, and older than 6 years of age). Group A had their surgical incisions sutured with polyglactin 10-0 material (Vicryl^®^, composed of polyglactin 910, 10-0 diameter, absorbable, a 0.62 cm, 3/8 circle needle) at the end of cataract surgery. Patients in Group B had their surgical incisions sutured with nylon 10-0 material (composed of nylon monofilament, 10-0 diameter, absorbable, a 0.55 cm, 1/2 circle needle) at the end of cataract surgery.

All cataract surgeries were performed by the same surgeon (M.V.M.) in accordance with the protocols used in the Department of Ophthalmology of the University of Campinas (UNICAMP). Preoperative antibiotics were not used. Briefly, the surgery protocol consisted of skin antisepsis with 10% povidone-iodine, placement of a sterile surgical drape to isolate lid margin and eyelashes, the application of four drops of 5% povidone-iodine into the conjunctival sac, and subsequent irrigation using a balanced salt solution. In cases of povidone-iodine allergy, an aqueous solution consisting of 0.05% chlorhexidine was used. Phacoaspiration using the Infiniti^®^ phacoemulsifier (Alcon Laboratories Inc., Fort Worth, Texas, USA) was performed, and an AcrySof^®^ MA60AC intraocular foldable lens (Alcon Laboratories Inc, Fort Worth, Texas, USA) was implanted in patients 6 months of age and older. Ultrasound energy was not used.

The Pediatric Cataract Department of the University of Campinas does not indicate intraocular lens implantation in patients younger than 6 months of age, and these patients remain aphakic. Intraocular lens implants are always recommended for patients 6 months of age and older. We chose to perform an a YAG laser posterior capsulotomy as an outpatient procedure; when patients were too young or otherwise unable to undergo the YAG procedure, we chose to perform a primary posterior capsulotomy (PPC) combined with an anterior vitrectomy (AV). With a few exceptions, patients 5 years of age and older exhibited the level of cooperation necessary to receive a YAG laser posterior capsulotomy.

We therefore performed one of three surgical combinations on each patient: on children younger than 6 months of age, we performed phacoaspiration combined with a PPC and an AV with no intraocular lens implant. In children 6 months of age and older who could not tolerate a YAG laser posterior capsulotomy, we performed phacoaspiration with an intraocular lens implant, as well as a PPC and an AV at the time of the cataract surgery. In children who were able to tolerate an outpatient YAG laser posterior capsulotomy, we performed phacoaspiration with an intraocular lens implant.

Clear corneal incision was used. Only one side port was made in all cases, and both incisions were sutured with the knots buried into the corneal side. The topical postoperative regimen consisted of 0.5% moxifloxacin combined with 0.1% dexamethasone every 3 h for 7 days only during waking hours. On the seventh postoperative day, this regimen was changed to only 0.1% dexamethasone, which was tapered over the course of 3 weeks.

The primary outcome was incidence of complications associated with sutures in each group. The suture-related complications were defined as corneal neovascularization close to the suture, loosening of the suture, accumulation of mucus on the suture, early suture rupture (within 2 weeks), aqueous humor leakage through the incision (as determined by the Seidel test), prolapse of the iris through the incision site, infectious or traumatic keratitis, endophthalmitis, and giant papillary conjunctivitis. In eyes with suture-related complications, the sutures were promptly removed. Secondary outcome was the need for suture removal under sedation in each group.

Sample size was calculated based on the frequency of suture-related complications described in the literature (15, 16) and using a two-tailed 95% confidence interval, 80% power, an exposed/unexposed radius of 1, and a null frequency of complications in the polyglactin suture group, which resulted in approximately 40 subjects per group. The trial would have been suspended if a difference between the two groups lower than a type I error (α) of 4% had been found in the pre-analysis of 50, 75, and 100% of the patients included. Eligible patients were randomly assigned at a 1:1 ratio. Randomization was stratified by age in block sizes of four. One nurse generated the random allocation sequence using a computer randomization list, and another nurse enrolled and assigned the subjects to the interventions in a masked fashion. Sealed opaque envelopes were used for allocation and were opened immediately before surgery.

After the attribution of the interventions, the patients and their guardians were masked to the type of intervention. The surgeons were not masked because the type of suture used can easily be identified during surgery and in the biomicroscopy exam in the postoperative period.

## Statistical Analysis

Continuous data have been expressed as the mean ± standard deviation and range. Medians and interquartile ranges were used for variables with non-normal distribution. Between-group differences of continuous variables were compared using the Wilcoxon signed-rank test, and categorical variables were compared using the *x*^2^ test or Fisher's exact test when appropriate. Multiple Firth logistic regressions were used to assess the effect of covariates on the studied outcomes. Analyses were performed using STATA 14.0 (StataCorp LP, College Station, TX, USA). Statistical significance was established when *p* ≤ 0.05.

## Results

**Figure 1:**
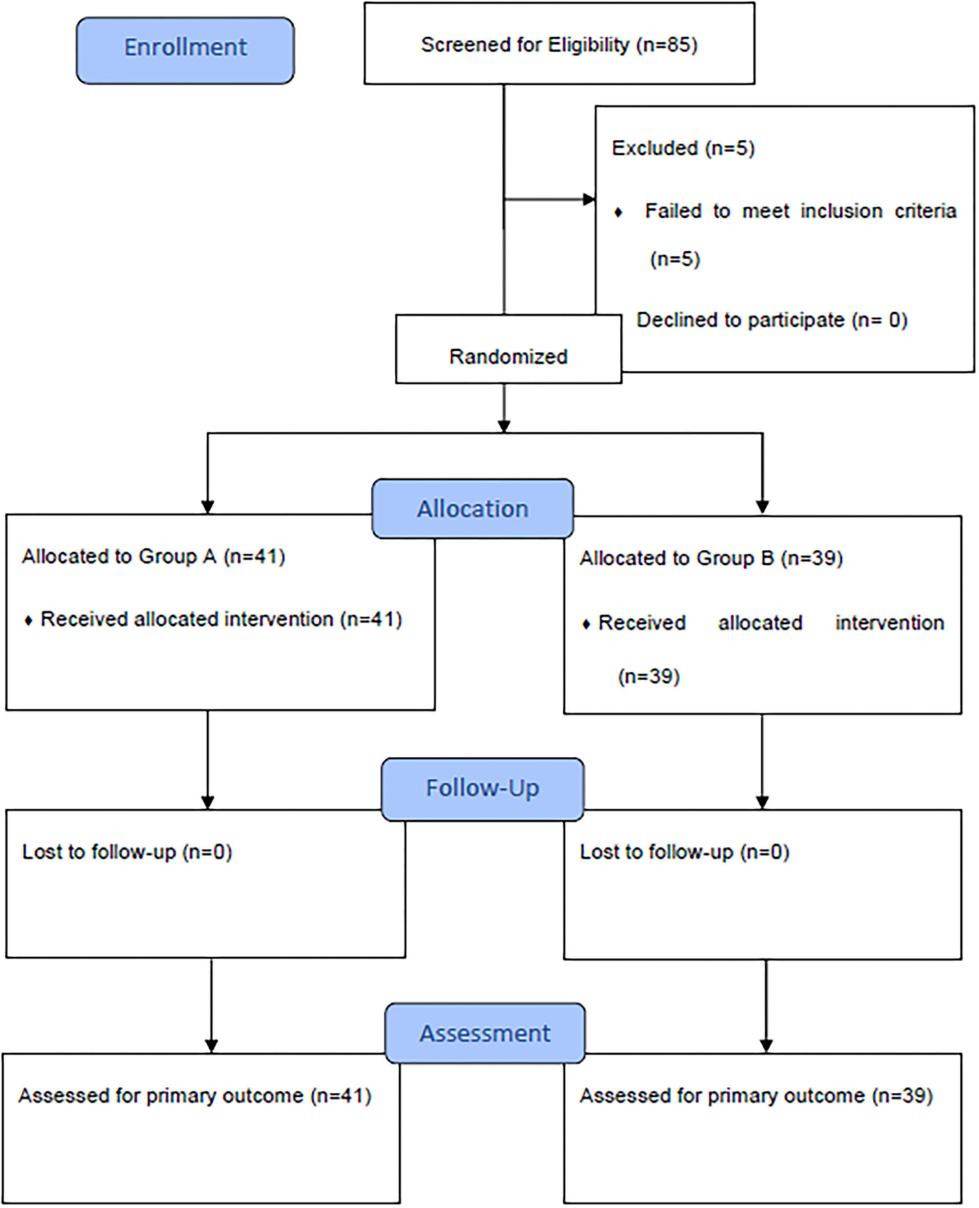
Comparison Between nylon and polyglactin corneal sutures in pediatric cataract surgery: CONSORT flow diagram.

This study enrolled 80 patients between January 2019, and August 2020. These subjects were randomized into group A (polyglactin suture) or group B (nylon suture). In total, 41 patients in the polyglactin group and 39 patients in the nylon group completed the 6 months of follow-up care; no subjects were lost to follow up (Figure 1). Demographic data demonstrate homogeneity between the groups, as displayed in Table 1.

**Table 1:** Demographic characteristics of pediatric cataract patients in the polyglactin suture group and the nylon suture group.

	**Polyglactin group**	**Nylon group**	***p*-Value**
**Sex**, *N* (%) **Male**	21 (51.22)	26 (66.67)	0.161^*^
**Age (months)**, mean ± SD (median; 25% percentile; 75% percentile)	45.76 ± 50.24 (16.00; 7.00; 72.00)	42.49 ± 46.41 (24.00; 5.00; 72.00)	0.780^†^
**Operated eye**, *N*(%) OD	18 (43.90)	25 (64.10)	0.070^*^
**Systemic comorbidities**, *N* (%)			0.151^*^
Marfan syndrome	3 (7.32)	7 (17.95)	
Toxoplasmosis	1 (2.44)	1 (2.56)	
Ichthyosis	1 (2.44)	1 (2.56)	
Galactosemia	0 (0.00)	3 (7.69)	
Down syndrome	1 (2.44)	2 (5.13)	
Cockayne syndrome	0 (0.00)	1 (2.56)	
No comorbidities	35 (85.37)	24 (61.54)	
**Ocular comorbidities**, *N* (%)			0.548^#^
Chorioretinitis scarring	1 (2.44)	0 (0.00)	
Persistent fetal vasculature	1 (2.44)	0 (0.00)	
Congenital glaucoma	1 (2.44)	1 (2.56)	
Strabismus	1 (2.44)	0 (0.00)	
Traumatic cataracts	0 (0.00)	1 (2.56)	
Aniridia	1 (2.44)	0 (0.00)	
No comorbidities	36 (87.80)	37 (94.87)	

*SD, Standard Deviation; OD, Right Eye*.

* *Chi-Squared test*.

† *Wilcoxon signed-rank test*.

# *Fisher's exact test*.

Table 2 shows the surgical options applied to each group.

**Table 2:** Pediatric cataract surgery outcomes organized by suture material used (polyglactin vs. nylon sutures).

	**Polyglactin group**	**Nylon group**	***p*-Value**
	***N* (%)**	***N* (%)**	
**Surgery type**			0.776^*^
Phaco + PPC + AV	23 (56.10)	25 (64.10)	
Phaco + PPC + AV + IOL	7 (17.07)	6 (15.38)	
Phaco + IOL	11 (26.83)	8 (20.51)	
**Intraoperative complications**			0.999^#^
PCR	1 (2.44)	1 (2.56)	
Iris damage	1 (2.44)	1 (2.56)	
None	39 (95.12)	37 (94.87)	
**Suture-related complications**			<0.001^#^
Vascularized suture	0 (0.00)	7 (17.95)	
Suture loosening	0 (0.00)	7 (17.95)	
Suture with mucus	0 (0.00)	1 (2.56)	
Suture rupture	0 (0.00)	2 (5.13)	
No complications	41 (100.00)	22 (56.41)	

*PPC, primary posterior capsulotomy; AV, anterior vitrectomy; IOL, intraocular lens; Phaco, phacoaspiration; PCR, posterior capsule rupture*.

* *Chi-Squared test*.

# *Fisher's exact test*.

Multiple logistic regression analysis was employed to determine the factors most closely associated with intraoperative complications, and the results are shown in Table 3.

**Table 3:** Multiple logistic regression analysis applied to data on intraoperative complications in pediatric cataract surgeries in which either nylon or polyglactin sutures were used.

	**Odds ratio (95% CI)**	***p*-Value**
**Sex**		0.178
Male	Reference	
Female	7.43 (0.40–137.25)	
**Age (months)**	1.02 (0.99–1.04)	0.062
**Systemic comorbidities**		0.232
No	Reference	
Yes	0.21 (0.02–2.69)	
**Ocular comorbidities**		0.820
No	Reference	
Yes	1.44 (0.06–32.22)	
**Surgery type**		-
Phaco + PPC + AV	Reference	
Phaco + PPC + AV + IOL	0.43 (0.02–10.86)	0.606
Phaco + IOL	0.55 (0.07–4.54)	0.578
**Type of suture material**		-
Nylon	Reference	
Polyglactin	2.04 (0.25–16.63)	0.504

*PPC, primary posterior capsulotomy; AV, anterior vitrectomy; IOL, intraocular lens; Phaco, phacoaspiration; CI, confidence interval*.

Table 4 shows the results of the multiple logistic regression employed to assess the factors associated with suture complications and the need for suture removal.

**Table 4:** Multiple logistic regression analysis applied to suture complications and the need for suture removal in cases of pediatric cataract surgeries with either nylon or polyglactin sutures.

	**Coefficient (95% CI)**	***p*-Value**
**Sex**		
Male	Reference	
Female	1.61 (0.38–6.86)	0.519
**Age (months)**	1.01 (0.99–1.03)	0.334
**Systemic comorbidities**		
No	Reference	
Yes	1.08 (0.23–5.17)	0.922
**Ocular comorbidities**		
No	Reference	
Yes	4.85 (0.29–81.94)	0.273
**Surgery type**		
Phaco + PPC + AV	Reference	
Phaco + PPC + AV + IOL	0.43 (0.05–3.54)	0.437
Phaco + IOL	0.13 (0.01–1.90)	0.136
**Intraoperative complications**		
No	Reference	
Yes	1.64 (0.21–12.86)	0.636
**Type of suture material**		
Nylon	Reference	
Polyglactin	61.69 (3.43–1110.93)	0.005

*PPC, primary posterior capsulotomy; AV, anterior vitrectomy; IOL, intraocular lens; Phaco, phacoaspiration; CI, confidence interval*.

## Discussion

Unlike in adult cataract surgery, pediatric cataract extraction surgery requires that the surgical incision be systematically sutured. In Brazil, the most commonly used suture material is currently nylon 10-0. Nylon is a monofilament and non-absorbable material composed of polyamides that enables increased suture tension time and induces minimal cellular reactions. Vicryl is an absorbable synthetic compound made of polyglactin (a copolymer of glycolide and lactide) (14). Suture absorption occurs as a result of hydrolysis, which itself is generated by glycolic and lactic acids. Many nylon sutures must be removed from patients during the postoperative period. Because the patients in these cases are children, this normally simple removal procedure requires sedation. As was demonstrated in our patient groups (Table 1), there is a major association between pediatric cataract and systemic diseases (Marfan syndrome, Down syndrome, congenital cardiac abnormalities, ichthyosis, inborn errors of metabolism, toxoplasmosis, rubella, syphilis, and other congenital infections). These comorbidities may increase the rate of complications in cases requiring general anesthesia in a group of patients whose age already makes them more vulnerable in this procedure (17, 18).

In addition to the risks associated with general anesthesia, suture removal itself also increases the risk of endophthalmitis in that it makes it easier for microorganisms to enter the eye as the string is being pulled (8). Certain measures must be taken to prevent this complication, including the use of 5% povidone-iodine before the procedure, cutting the suture at one end to prevent the external portion of the string from entering the eye, postoperative topical antibiotics, and outpatient follow-up care to catch and treat any infections as early as possible.

Other researchers have also compared different suture materials for use in pediatric cataract surgery. Bar-sela et al. (15). performed a retrospective study to compare polyester 10-0 (Mersilene®) to polyglactin 10-0 (Vicryl®) over 6 months of follow up and, similar to our results, found no suture-related complications in the polyglactin 10-0 group. Matalia et al. performed a non-randomized prospective study to compare the same suture materials considered herein in pediatric cataract surgeries and obtained similar results (16). As in the surgeries performed herein, these researchers found the polyglactin string to be more difficult to work with and bury. In Matalia et al. and in our study, this finding is merely anecdotal and was not measured quantitatively.

Sukhija and Kaur (20) performed a non-randomized prospective study to compare the efficacy and outcome of viscosealing the incisions using 1.4% sodium hyaluronate with polyglactin sutures in children <5 years. They concluded viscosealing is comparable to suturing of incisions in children undergoing cataract surgery.

Bartholomew et al. (19) also performed a prospective randomized study to compare polyglactin 8-0, nylon 10-0, and silk 8-0, but they reported a greater risk of complications in the polyglactin group within the first month after surgery relative to their other study groups. This finding differs from more recent studies because, according to the authors, they experienced difficulty in knotting the polyglactin string, which resulted in ineffective incision closure. It is important to note that their study was performed in 1976, before the advent of phacoemulsification; therefore, the number of sutures required to close the incision was considerably higher than is required today. This large number of sutures predisposed patients to experiencing suture-related complications. Furthermore, this study was performed on adult patients.

As in other well-known studies on pediatric patients (15, 16), this study concluded that polyglactin 10-0 (Vicryl®) sutures were associated with fewer complications than polyester 10-0 (Mersilene®) sutures; the results therefore demonstrate the benefits of this polyglactin product. Our study seems to be the first to compare different suture materials in prospective and randomized groups of children. We found that the only factor associated with suture-related complications in and following pediatric cataract surgery was the type of material used: subjects who received the nylon sutures were 61.7 times more likely to experience suture-related complications and to require suture removal relative to subjects who received polyglactin sutures (OR = 61.69; 95% CI: 3.43–1110.93; *p* = 0.005). The most frequent complications were vascularization near the suture (17.95%) and suture loosening (17.95%). In the nylon group, the mean time for suture removal was 88.23 ± 59.50 days (median = 60 days). It is important to note that there were no significant differences between the groups in terms of types of surgery (*p* = 0.776) or intraoperative complications (*p* = 0.776).

This study presents some limitations. The degree of astigmatism induced was not assessed due to the lack of adequate methods available for measurement in children, who are unable to undergo in-office keratometry. The exact amount of time required for polyglactin absorption was not determined; however, at the end of the 6-month follow-up period, the biomicroscope exams performed on the polyglactin group revealed no evidence of any suture remains. Our study would have been improved by data comparing the average amount of time spent on suturing in each of the groups, since, as mentioned previously, we experienced greater difficulty and required more time to suture with polyglactin string than with nylon. In a future study, we plan to assess the cost-effectiveness of the use of polyglactin string: though this material is more expensive than nylon, it clearly reduces the need for both additional procedures and the use of general anesthesia for suture removal.

Our study is the first randomized clinical trial to demonstrate that absorbable polyglactin 10-0 sutures are safe for use in pediatric cataract surgery and result in fewer postoperative complications than non-absorbable nylon 10-0 sutures. None of the patients who received polyglactin sutures required suture removal under sedation; these patients were therefore spared the risks associated with general anesthesia and with suture removal itself.

## Data Availability Statement

The raw data supporting the conclusions of this article will be made available by the authors, without undue reservation.

## Ethics Statement

The studies involving human participants were reviewed and approved by Ethics committee of the School of Medical Sciences, University of Campinas (UNICAMP). Written informed consent to participate in this study was provided by the participants' legal guardian/next of kin.

## Author Contributions

MM, RL, CA, and MA contributed to conception and design of the study. MM and RR organized the database. MM, RR, DO, and MA performed the statistical analysis. MM and MA wrote the first draft of the manuscript. MM, RR, RL, DO, CA, and MA wrote sections of the manuscript. All authors contributed to manuscript revision, read, and approved the submitted version.

## Conflict of Interest

The authors declare that the research was conducted in the absence of any commercial or financial relationships that could be construed as a potential conflict of interest.

## Publisher's Note

All claims expressed in this article are solely those of the authors and do not necessarily represent those of their affiliated organizations, or those of the publisher, the editors and the reviewers. Any product that may be evaluated in this article, or claim that may be made by its manufacturer, is not guaranteed or endorsed by the publisher.
